# Theoretical insights into site-specific heavy-atom effects on MR-TADF emitters: modulation of spin–orbit coupling and color purity

**DOI:** 10.1039/d6sc00582a

**Published:** 2026-03-20

**Authors:** Shi-Jie Ge, Jian-Rong Wu, Zuo-Quan Jiang

**Affiliations:** a State Key Laboratory of Bioinspired Interfacial Materials Science, Institute of Functional Nano & Soft Materials (FUNSOM), Soochow University Suzhou 215123 Jiangsu PR China zqjiang@suda.edu.cn

## Abstract

Introducing heavy atoms is a promising strategy to enhance triplet-to-singlet upconversion in multi-resonance thermally activated delayed fluorescence (MR-TADF) materials. By bolstering spin–orbit coupling (SOC), this approach suppresses triplet–triplet annihilation and consequently alleviates efficiency roll-off in devices. However, the embedded heavy atoms at different sites often exert a dual influence on both the SOC and the color purity. This study systematically investigates three types of heavy-atom-embedded MR-TADF emitters derived from a QAO core, with modifications at the bay position, on a spiro-fused periphery, and through a noncovalent face-on interaction. The high-level quantum chemical calculations reveal that the enhancement of SOC strongly depends on the heavy atom's position relative to the luminescent core. Direct embedding into the luminescent core significantly increases SOC but causes red-shifted and broadened spectra. In contrast, placing heavy atoms farther from the luminescent core diminishes their effect. Notably, non-covalent incorporation of heavy atoms into the luminescent core can balance increasing SOC while preserving a narrow full width at half maximum (FWHM). This investigation provides theoretical insights for designing efficient MR-TADF materials which simultaneously suppress efficiency roll-off and achieve high color purity.

## Abstract 2

In multi-resonance thermally activated delayed fluorescence (MR-TADF) materials, the introduction site of heavy atoms exhibits an antagonistic effect on color purity and spin–orbit coupling.

## Graphical abstract

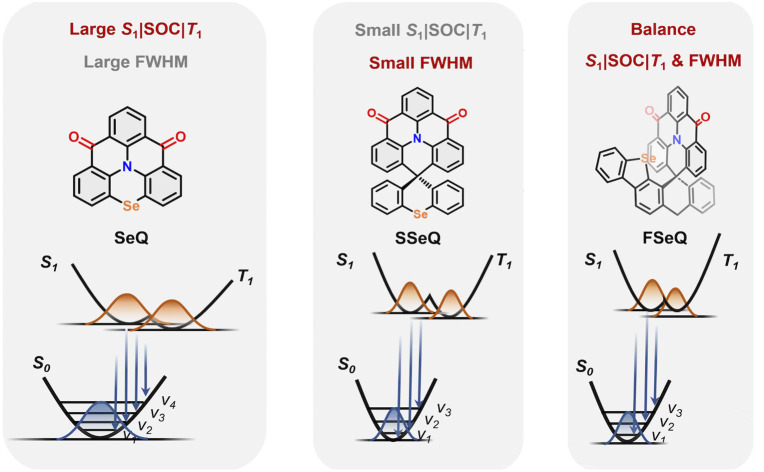

## 1 Introduction

Recently, multi-resonance thermally activated delayed fluorescence (MR-TADF) has attracted considerable attention in the field of organic light-emitting diodes (OLEDs) due to its high luminous efficacy and excellent color purity.^1–7^ In 2016, Hatakeyama *et al.* first designed an *ortho*-substituted graphene fragment structure doped with boron and nitrogen atoms (DABNA-1), which ingeniously achieves an atomic-scale spatial separation between the highest occupied molecular orbital (HOMO) and the lowest unoccupied molecular orbital (LUMO).^8^ From a quantum chemical perspective, this separation reduces the exchange energy (*K*_HL_) between HOMO and LUMO, corresponding to a decrease in the spin-dependent energy difference. As a result, the singlet–triplet energy gap (Δ*E*_ST_) is minimized, thereby facilitating the reverse intersystem crossing (RISC) process of triplet excitons.^9–11^

The RISC process and high color purity represent critical characteristics for the practical application of MR-TADF materials. On the one hand, a higher RISC rate (*k*_RISC_) shortens the triplet exciton lifetime, thereby suppressing efficiency roll-off at high concentration or brightness caused by triplet–triplet annihilation. This, in turn, enhances device efficiency and prolongs the operational lifetime of OLEDs.^12–15^ On the other hand, unlike conventional donor–acceptor (D–A) TADF materials, which exhibit long-range charge transfer (LRCT) excited states, MR-TADF molecules with fused-ring scaffold exhibit short-range charge transfer (SRCT), where charge redistribution occurs only between adjacent atoms, resulting in a narrow and steep potential energy surface of the electronic states. In addition, the rigid molecular skeleton suppresses vibrational coupling, nonradiative decay, and spectral broadening, leading to emission spectra with distinct structural features and narrow bandwidths.^16–22^ Consequently, research on MR-TADF has mainly focused on two key aspects: (i) enhancing the RISC process of triplet excitons, and (ii) improving the color purity of emissive materials.

Researchers have adopted various strategies to enhance the *k*_RISC_ rate, including: (i) extending the π-conjugated system to reduce Δ*E*_ST_;^23–25^ (ii) exploiting the El-Sayed rule by configuring distinct singlet–triplet transition pathways, such as introducing different proportions of LRCT character, mixing π–π* and *σ*–π* transitions;^5,26–28^ and (iii) employing the heavy-atom effect to increase spin–orbit coupling (SOC) between singlet and triplet states.^2,29–34^ Among these, the third approach allows the incorporation of heavy-atom into MR-TADF materials, leading to the design of oxygen- (O), sulfur- (S), and selenium- (Se) containing molecules. Remarkably, when progressing from O to S and then to Se, the *k*_RISC_ rate can be enhanced by one to two orders of magnitude (10–50 times). This trend arises because *k*_RISC_∝|〈S|*Ĥ*_SO_|T〉|^2^ in the Marcus rate, and the spin–orbit Hamiltonian in multi-electron systems, expressed as:^35,36^1
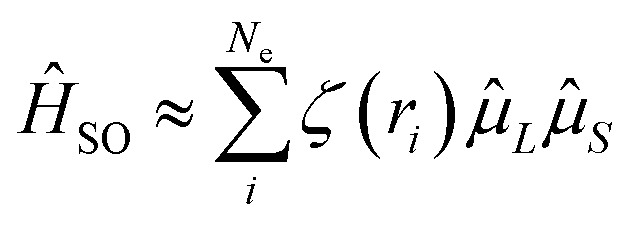
where *ζ*(*r*_*i*_) denotes the SOC potential experienced by electron *i* at position *r*_*i*_, originating from relativistic effects as the electron moves within the Coulomb potential field of the nucleus. *L* and *S* represent the orbital and spin angular momenta, while μ̂*L* and μ̂*S* correspond to the associated magnetic moments. Because heavy atoms possess larger nuclear charges (Ze), the resulting Coulomb field is stronger, thereby yielding more pronounced SOC effects.

However, numerous experimental studies have reported that direct embedding heavy atoms such as S or Se into the luminescent core of MR-TADF molecules often induces a red-shift and significant broadening of the emission spectra, thereby severely compromising color purity.^29,37^ Alternatively, a balance must be struck between enhancing SOC *via* heavy atoms and preserving the color purity of the emitter. This necessitates precise control over the substitution sites of heavy atoms and rational molecular design to simultaneously maintain the emission wavelength and narrowband characteristics, while only moderately increasing the *k*_RISC_ rate. For example, Yang and co-workers achieved *k*_RISC_ rates on the order of 10^5^–10^6^ s^−1^ while maintaining full width at half maximum (FWHM) values below 30 nm by incorporating Se atoms into peripheral substituents or the outer framework of MR-TADF cores.^24^ Our group has demonstrated that employing spiro-structured spatial effects can indirectly enhance heavy-atom-induced SOC through noncovalent interactions, yielding a *k*_RISC_ enhancement together with a narrow FWHM of 24 nm.^34^ These results highlight the critical importance of “site selectivity” and “interaction strength modulatio” in heavy-atom modification strategies, providing effective solutions for balancing SOC enhancement with the retention of spectral purity. Quantum chemical calculations serve as powerful tools to interpret and guide experiments. In-depth theoretical analyses of these phenomena are essential to shed light on the design of highly efficient narrowband MR-TADF materials.

**Fig. 1:**
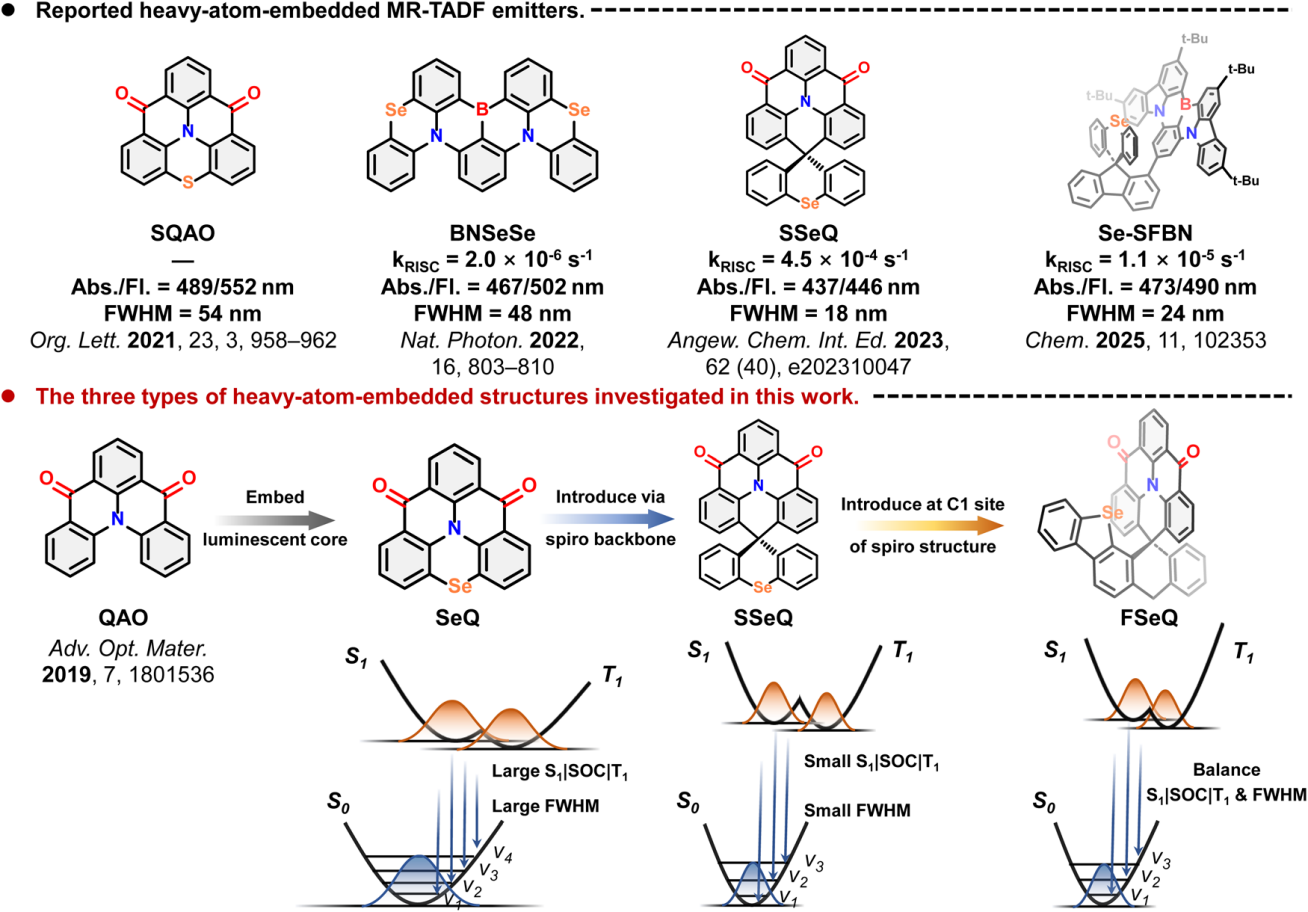
Reported heavy-atom-embedded MR-TADF emitters and the three types of heavy-atom-embedded structures investigated in this work.

In this work, we conducted high-level quantum chemical calculations to systematically investigate three types of heavy-atom-embedded luminescent materials derived from the QAO core, as shown in Fig. 1.^38^ These types include: (i) incorporation of heavy-atom directly into the bay positions of the luminescent core (SeQ); (ii) introduction of heavy atoms through a spiro-based scaffold (SSeQ); and (iii) substitution of heavy-atom at the C1 position of the spiro unit, where they interact with the luminescent core in a noncovalent manner (FSeQ). Meanwhile, a comparative study was also conducted by embedding O and S elements. Although these three modification strategies have been experimentally reported, in-depth theoretical analyses remain scarce. Herein, we focus on elucidating the role of heavy atoms at different modification sites in enhancing SOC, and their influence on the photophysical process.

## 2 Computational methods

To accurately describe both the ground-state orbital band gaps and the excited states of the studied systems, we employed the optimal-tuned screened range-separated hybrid functional in combination with a polarized continuum model (OT-SRSH-PCM).^39,40^ This approach accounts for electron exchange–correlation interactions while consistently coupling long-range corrections with dielectric polarization effects, thereby enabling an accurate description of the electronic structure in solvated systems. The range-separated hybrid functional partitions the electron–electron interaction into distance-dependent exchange contributions, significantly improving the description of long-range interactions:^41–43^2
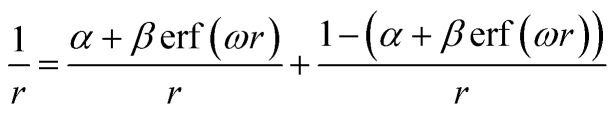
where *r* is the electron-interaction distance, and *α*, *β*, and *ω* are tunable parameters. The first term is evaluated using exact (Fock) exchange, whereas the second term is described by a semilocal exchange functional. In the gas phase, where dielectric screening is absent, *α* + *β* = 1. The optimal tuning (OT) procedure determines the switching parameter *ω* between the short- and long-range regimes with a fixed *α* value, by minimizing a target function *J*^2^(*ω*):

3 *J*^2^(*ω*) = [*ε*_HOMO_(*ω*) − *E*_IP_(*ω*)]^2^ + [*ε*_A_HOMO_(*ω*) + *E*_EA_(*ω*)]^2^

where *ε*_HOMO_ and *ε*_A_HOMO_ are the eigenvalues of the HOMO of the neutral molecule and the HOMO of the anionic system, respectively, and *E*_IP_ and *E*_EA_ are the ionization potential and electron affinity energies. In the SRSH-PCM scheme, *α* + *β* ≡ 1/*ε*, where *ε* is the dielectric constant of the solvent. In this way, dielectric screening of long-range electrostatic interactions is treated consistently across both components of the SRSH-PCM framework.

Using density functional theory (DFT) and time-dependent (TD-) DFT, ground- and excited-state calculations were carried out at the LC-BLYP/def2-SVP level of theory with the PCM model in cyclohexane (*ε* = 2.02) using the Gaussian16 package.^44–47^ LC-BLYP combines 100% long-range exact exchange with pure DFT exchange in the short range, offering a non-empirical description of asymptotic exchange. This avoids the empirical tuning of short-range Hartree–Fock exchange (HFx) fractions found in functionals like CAM-B3LYP (19–65% HFx) and *ω*B97X-D (22–100% HFx).^48,49^ The def2 basis sets utilize all-electron basis sets (no pseudopotentials) for Se element. Molecular orbital analysis, excited state analysis, and independent gradient model based on Hirshfeld partition (IGMH) analysis were performed using Multiwfn 3.8 dev (update 2025-Jun-3) and visualized with VMD 1.9.4 software.^50–52^ The sobEDA software used for energy decomposition analysis.^53^

The vibrationally resolved decomposition of reorganization energy (*λ*) between the S_0_ and S_1_ was evaluated as:4
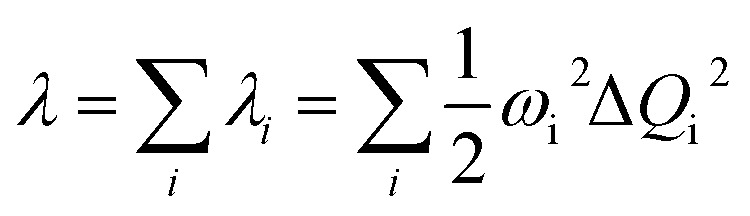
where *λ*_*i*_ denotes the reorganization energy associated with the *i*-th vibrational mode with frequency *ω*_*i*_, and Δ*Q*_*i*_ represents the mass-weighted normal coordinate displacement of the corresponding vibrational coordinate between the equilibrium geometries of the two electronic states involved.

## 3 Results and discussion

### 3.1 Computational results for OQ, SQ, SeQ

**Fig. 2:**
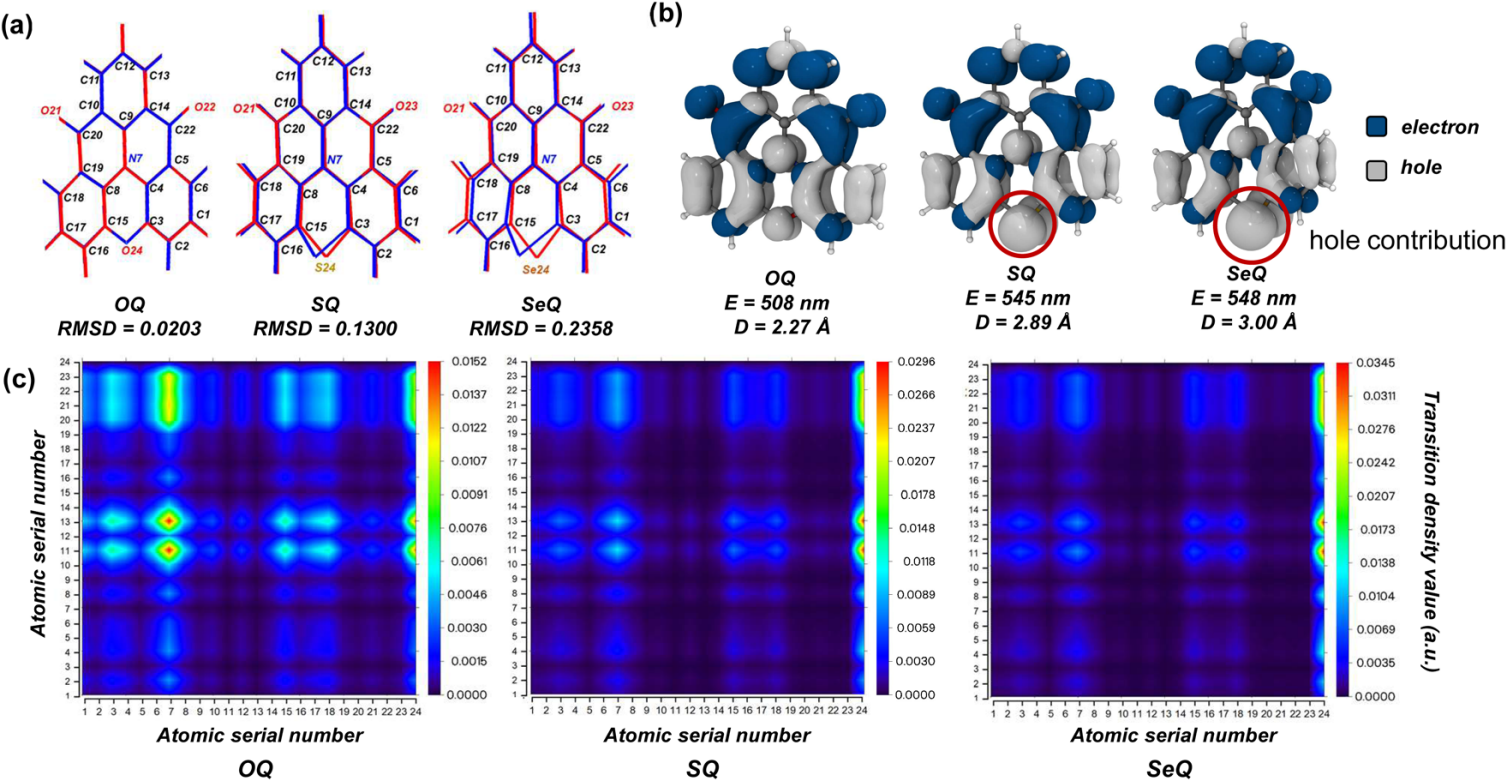
(a) The geometric difference between the S_0_ (blue) and S_1_ (red) configurations as well as atomic serial number, (b) electron-hole distribution of S_1_ state (E denotes energy, and D represents the centroid distance between electrons and holes) , and (c) S_1_ state transition density matrix heat maps of OQ, SQ, and SeQ.

Firstly, we focus on the modification strategy involving the direct embedding of heavy atoms into the MR-TADF emitting core. In 2019, after the report of the MR-TADF material QAO characterized by an N/C=O motif, it was observed that the bay position suffered from H⋯H steric repulsion, which introduced a dihedral twist.^38^ This significantly increased the molecular flexibility and was detrimental to the emission properties. To address this, subsequent studies substituted the bay-position C–H bonds with heavy atoms integrated into the π-conjugated framework (OQ, SQ, and SeQ), thereby enforcing a rigid planar geometry (Fig. 2a).^37,54^ However, the resulting molecules all exhibited varying degrees of red-shifted and broadened emission spectra in experiments. For fluorescent molecules, such behavior is mainly determined by the nature of the ground state (S_0_) and excited state (S_1_). Therefore, we first performed geometric optimizations for S_0_ and S_1_ states. The optimized structures are shown in Fig. 2a, S1 and S2. Notably, in the S_0_ state, the C–S–C and C–Se–C units display pronounced out-of-plane bending, with Se exhibiting an even larger deviation. In contrast, in the S_1_ state, the C–X–C fragments of all three molecules relax into coplanarity with the fused-ring framework, highlighting the crucial role of heavy atoms in excited-state structural relaxation. By comparison, the lighter O atom induces negligible structural displacement in the excited state, with the root means square deviation (RMSD) value changing by only 0.02. In contrast, SQ and SeQ display substantially larger RMSD increases to 0.13 and 0.24, respectively.

To elucidate the conformational changes in the excited state, we turn to the electronic structures. The MR-TADF luminescent core is governed by the delocalization of π electrons through multiple resonance interactions between donor (D) and acceptor (A) atoms within the fused-ring scaffold. The degree of spatial overlap and energy alignment of the involved orbitals are key factors determining the strength of resonance effects. For N, C, and O atoms, their 2p orbitals participate in π delocalization, resulting in strong electron delocalization and resonance effects. Consequently, the coplanarity of the C–O–C fragment with the fused ring is maintained. By contrast, heavy atoms such as S and Se possess valence electrons in higher-energy orbitals. When incorporated into the fused ring, the 3p orbital of S can still overlap with the 2p orbitals of the π system, whereas the 4p lone pair of Se remains largely decoupled from π conjugation. The Se atom is forced out of the molecular plane by the repulsion from its lone pair electrons against the adjacent π-system. This structural distortion ultimately reflects the atom's preference for sp^3^ (tetrahedral) over sp^2^ (planar) hybridization. It is worth noting that the reported single-crystal structure of SQ displays a planar geometry, in contrast with our optimized results.^37^ This discrepancy arises because intermolecular interactions in the crystal lattice compress the molecule into planarity to lower the overall energy, whereas in our simulations mimicking solution-phase conditions, the molecules are free to relax without such constraints.

Further electron-hole analysis provides additional insight into the influence of heavy atoms on the excited states. For all three molecules, the S_1_ state is primarily governed by HOMO–LUMO transitions. Due to the presence of S and Se, the HOMO levels are destabilized (elevated energy level), while the LUMO levels remain largely unaffected. As a result, SQ and SeQ possess lower S_1_ energies than OQ, and their vertical excitation wavelengths are red-shifted to around 480 nm relative to OQ (Table S2). Upon structural relaxation in the excited state, consistent with the earlier analysis, the larger RMSD values of SQ and SeQ lead to reorganization energies of 0.27 and 0.32 eV, respectively, and enhanced Stokes shifts. Consequently, their emission wavelengths are further red-shifted to 545 and 548 nm, in good agreement with experimental reports.^37,54^ As shown in Fig. 2b, contributions of O, S, and Se to the hole distribution increase sequentially, leading to a larger centroid distance between electrons and holes and thus enhancing the LRCT character in the excited states. To provide a more detailed picture of the electron-hole distributions, we performed transition density matrix analysis. As illustrated in Fig. 2c, in OQ, the charge transfer predominantly occurs from atoms N7, C3, C15, C18, C6, and O24 to atoms C11, C13, C20, O21, O22, and C22, with distinct intensity. Specifically, the electron-rich N atom and the electron-deficient carbonyl groups govern the MR charge-transfer distribution. Moreover, the benzene ring containing C12 and C13 is strongly influenced by the adjacent carbonyls, while the benzene ring containing C6 and C18 is affected by the donor groups N7 and O24. These contributions clearly highlight the multiple-resonance effect in OQ. In SQ and SeQ, the transition density matrices show qualitatively similar distributions but with altered intensities. SRCT dominated by N/C=O is attenuated, whereas LRCT involving the bay-position S/Se atoms is enhanced, suggesting that the MR effect is gradually weakened in these two molecules.

**Fig. 3:**
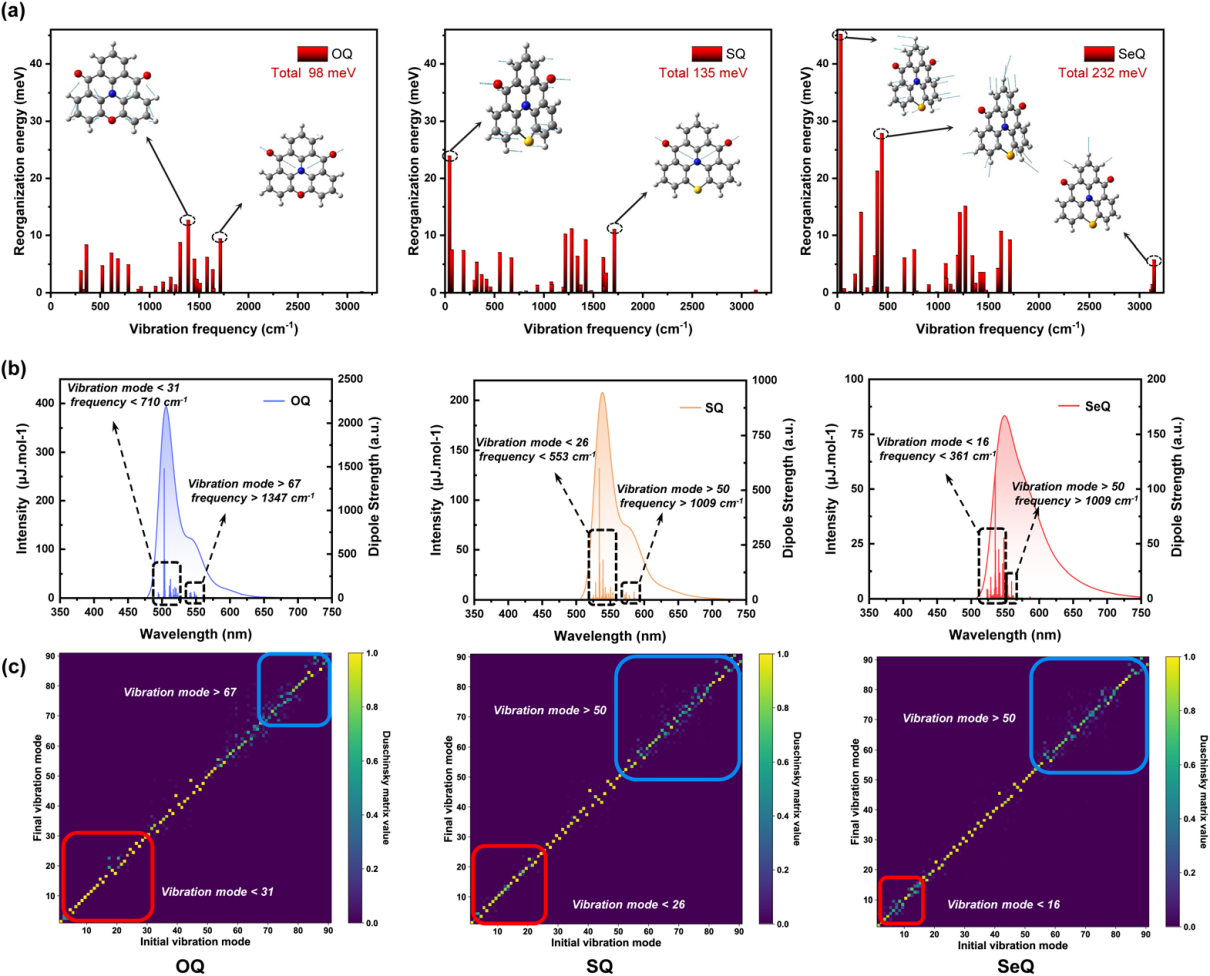
(a) Vibrationally resolved decomposition of reorganization energy (*λ*), (b) vibrationally resolved electron emission spectra with vibronic coupling transitions, and (c) heatmap of the Duschinsky matrix illustrating the coupling between excited state (initial) and ground (final) vibration modes of OQ, SQ, and SeQ.

MR-TADF emitters are characterized by their narrowband spectra. Given that the broadening of luminescence spectra is closely related to the reorganization energy and molecular vibration, we further performed vibrationally resolved decomposition calculations of *λ* between the S_0_ and S_1_ states in Fig. 3a. By decomposing *λ* into individual vibrational modes, we were able to pinpoint the vibrational modes responsible for the observed spectral profile. As shown in Fig. 3a, mode-resolved *λ* analysis reveals that in OQ the reorganization energy mainly originates from vibrations in the 1000–1700 cm^−1^ region, arising from C=C bending and C=O stretching modes. By contrast, SQ exhibits a significantly larger *λ*, with the additional contribution predominantly from low-frequency vibrations below 500 cm^−1^. These vibrations are associated with skeletal motions of the entire molecular scaffold, indicating that molecular flexibility is markedly enhanced. For SeQ, both low- and high-frequency modes contribute more substantially to the reorganization energy, suggesting that excited-state electronic redistribution induces extensive structural rearrangements involving local units as well as the global framework, leading to larger Δ*Q* values across vibrational modes. This observation is consistent with the transition density matrix analysis, which indicated enhanced LRCT in SeQ.

As shown in Fig. 3b, the calculated vibronically resolved spectra further illustrate the impact of heavy-atom modulation on the spectral lineshape. Using TD-DFT in combination with the Duschinsky rotation matrix approach, we simulated the vibrational fine structure associated with the S_0_ → S_1_ electronic transition, where the Franck-Condon approximation was applied to treat electron-vibration coupling. As shown in the simulated spectra, OQ exhibits a relatively weak shoulder peak and a narrow FWHM. The analysis of the electron-vibration coupling transition shows that near the main peak transition region, the spectral characteristics are mainly dominated by the coupling of low-frequency vibration modes (mode < 31). High-frequency vibration (mode > 67) coupling causes electrons to fall back to the high-frequency vibration state of the ground state, resulting in the emission energy being lower than the main peak transition, which is manifested as redshift. In SQ, the enhanced low-frequency contributions lead to stronger vibronic coupling near the main peak transition, thereby broadening the FWHM, whereas the high-frequency contributions are less influential. When focusing on SeQ, the shoulder and the main peak merge, resulting in a broad emission spectrum. It exhibits a dramatic increase in both low- and high-frequency contributions, producing an intense shoulder that merges with the main peak, yielding a broadened spectrum devoid of distinct fine structure. In Fig. 3c, the heatmap of the Duschinsky matrix clearly illustrates the coupling between vibrational modes of the excited and ground states. When the signals are concentrated along the diagonal, the vibrational modes of the two states are essentially preserved, and the Franck-Condon factors are dominated by a few key modes. In contrast, the presence of pronounced off-diagonal features indicates mode rotation and mixing, where a single excited-state vibrational mode projects onto multiple ground-state modes. From OQ to SQ and SeQ, the degree of decoupling in low-frequency modes gradually increases, with stronger asymmetric signals, whereas the high-frequency modes show slightly reduced mixing. Although the mid-frequency region also exhibits noticeable off-diagonal signals in the Duschinsky matrix, these correspond mainly to minor bending or stretching motions with only small geometric displacements. As a result, their contribution to the reorganization energy is limited, leading to weak electron-vibration coupling and negligible intensity in the spectra. These results suggest that to achieve highly color-pure emission, two strategies are essential: (i) the suppression of local high-frequency vibrational modes responsible for intense shoulder peaks, for example by avoiding functional groups that introduce high-frequency vibrations; and (ii) maximizing molecular rigidity to reduce the activity of low-frequency skeletal vibrations, thereby minimizing vibronic coupling near the main transition and generating sharper emission profiles.

### 3.2 Computational results for SOQ, SSQ, SSeQ

In QAO, embedding heavy-atom at the bay position introduces partial LRCT, which enhances molecular flexibility and leads to spectral broadening. To overcome this drawback, subsequent studies adopted alternative modification strategies. Among them, spiro scaffold, owing to their unique stereochemical configuration and rigid structure, have been widely utilized in organic optoelectronics. In 2023, our group proposed a spiro-locked strategy for constructing a series of N/C=O-based MR-TADF cores, in which heavy atoms were incorporated into one side of the spiro scaffold, yielding SOQ, SSQ, and SSeQ.^55^ Molecular orbital calculations revealed that the embedded S and Se atoms elevate the orbital energy levels of the spiro fragment, rendering them frontier orbitals. Because the electronic transitions occur between spatially proximate orbitals, the transition dipole moments become large, and the SRCT states undergo configuration interaction, thereby lowering their excitation energy. As a result, the S_1_ state is reorganized into a HOMO−1 → LUMO transition while maintaining the MR-TADF characteristics (Table 1 and Fig. S13).

**Table 1:** The S_1_ properties obtained from TD-DFT calculations

Molecule	*E* a (eV)	*f* b	*D* c (Å)	*S*_r_ d (a.u.)	*t*_idx_ e	orb^f^	〈S|*Ĥ*_SO_|T〉|^2^(cm^−1^)
OQ	2.44	0.17	2.27	0.61	0.32	HOMO → LUMO: 0.983	0.00
SQ	2.28	0.12	2.86	0.55	0.89	HOMO → LUMO: 0.975	0.06
SeQ	2.26	0.10	3.01	0.53	1.01	HOMO → LUMO: 0.975	78.45
SOQ	2.81	0.15	1.87	0.62	−0.16	HOMO → LUMO: 0.975	0.00
SSQ	2.85	0.15	1.85	0.62	−0.19	HOMO−1 → LUMO: 0.974	0.01
SSeQ	2.86	0.15	1.84	0.62	−0.20	HOMO−1 → LUMO: 0.974	0.13
FOQ	2.75	0.13	2.00	0.61	0.00	HOMO → LUMO: 0.977	0.17
FSQ	2.79	0.14	1.92	0.62	0.22	HOMO → LUMO: 0.976	0.41
FSeQ	2.81	0.14	1.88	0.62	0.24	HOMO → LUMO: 0.975	3.24

a Energy level.

b Oscillator strength.

c Centroid distance between electron and hole.

d Hole–electron overlap integral.

e Charge transfer index.

f Orbital composition.

**Fig. 4:**
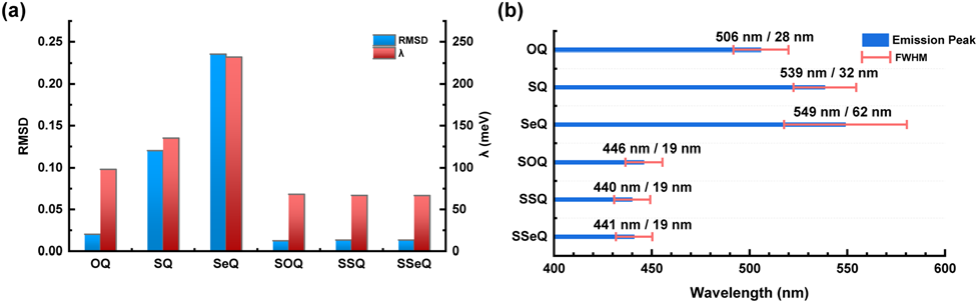
(a) The RMSD values and *λ* and (b) comparison of emission peak and FWHM of OQ, SQ, SeQ, SOQ, SSQ, and SSeQ.

The sp^3^ carbon atom at the spiro center imparts strong rigidity to the molecular geometry. Consequently, the calculated RMSD and reorganization energies of the three molecules are significantly reduced compared to those with heavy atoms embedded directly at the bay positions (Fig. 4a). The calculated parameters indicate that the three molecules exhibit similar excited-state configurations. This conclusion is further confirmed by transition density matrix analysis, which reveals that the nitrogen atom, the carbonyl group, and the resonance-stabilized benzene carbons are the primary contributors to the SRCT (Table 1 and Fig. S16). In addition, spiro carbons C27 and C32 participate in the emission core *via σ*–π hyperconjugation, resulting in minor charge transfer contributions. Apart from this, the spiro-substituted fragment does not contribute substantially to the excited states. This indicates that the three molecules possess nearly identical photophysical properties. Vibronically resolved spectra further corroborate this conclusion, showing consistent electronic-vibrational coupling transitions across the series. Compared with OQ, SQ, and SeQ, the spectra of the spiro derivatives exhibit a distinct blue shift and narrowing, highlighting the superiority of the spiro framework for molecular design in organic emitters (Fig. 4b).

However, experimental studies have shown that in the solid state, all three molecules exhibit Δ*E*_ST_ of 0.21 eV, with *k*_RISC_ on the order of 10^4^ s^−1^, suggesting that the introduction of heavy atoms through the spiro backbone does not significantly enhance SOC. Indeed, analysis of the SOC constant between the lowest singlet and triplet states revealed that 〈S_1_|*H*_SO_|T_1_〉 for SSeQ is merely 0.13 cm^−1^, which is far smaller than the 78.45 cm^−1^ observed in SeQ with direct bay-position embedding (Table 1). Structurally, in SSeQ the selenium atom resides at a non-conjugated position within the spiro fragment, spatially separated from the π-conjugated luminescent core, thereby strongly attenuating its SOC effect. In contrast, in SeQ the selenium atom is close to the core, enabling more efficient SOC transfer *via* the adjacent orbitals. Although higher triplet states (T_2_, T_3_) have been reported to participate in RISC, Boltzmann statistics dictate that the population of higher-energy states is significantly lower. Thus, the T_1_ → S_1_ process remains critical to TADF performance.

### 3.3 Computational results for FOQ, FSQ, and FSeQ

Noncovalent interactions, which do not involve the formation of chemical bonds, are primarily stabilized by weak intermolecular or intramolecular forces. In recent years, such interactions have found broad applications in the field of organic materials.^56–61^ In 2025, our group further proposed a strategy to enhance MR-TADF emission *via* spatial heavy-atom effects. By introducing heavy atoms into the MR-TADF molecules through both “intramolecular and external heavy-atom effects”, our group established short-range spatial interactions with the chromophore rather than extending the conjugation pathway.^34,62^ This modification enhanced the rate of reverse intersystem crossing (*k*_RISC_) while preserving the spectral characteristics of the MR chromophore. Taking spiro-modified N/C=O systems as examples, heavy atoms were introduced at the C1 site of the spiro moiety. Theoretical investigations were conducted to explore the role of spatial heavy-atom effects, providing insights and guidance for their implementation.

**Fig. 5:**
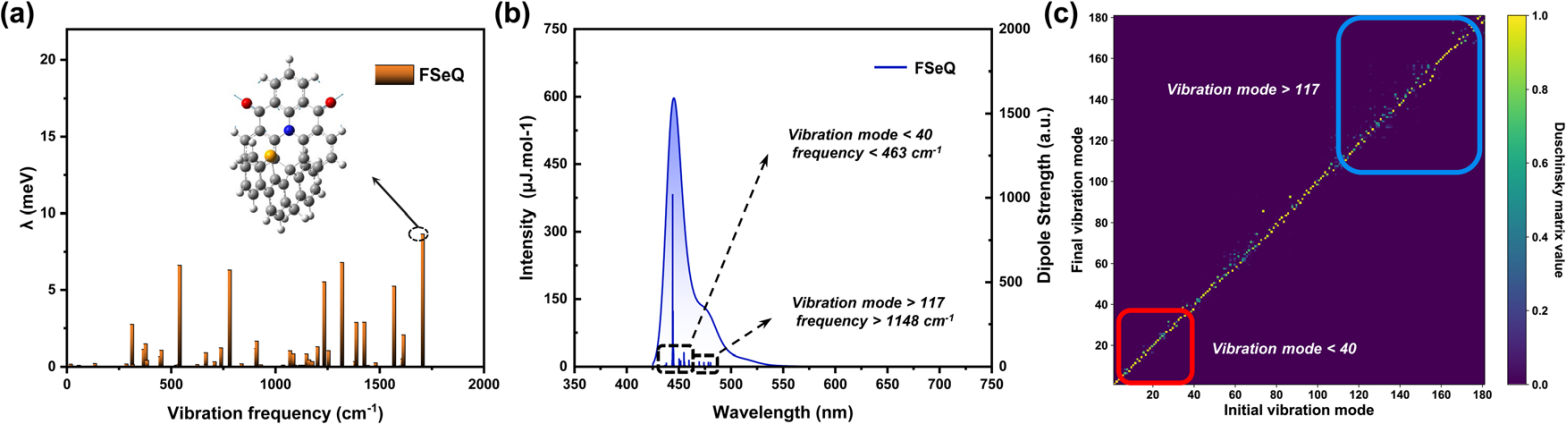
(a) Vibrationally resolved decomposition of *λ*, (b) vibrationally resolved electronic spectra along with different modes of vibronic coupling transitions, and (c) heatmap of the Duschinsky matrix illustrating the coupling between the excited state (initial) and ground (final) vibration modes of FSeQ.

Firstly, the substitution at the C1 position of the spiro scaffold introduces steric hindrance, shifting the emission core toward the opposite side. This steric effect intensifies with increasing atomic radius of the atom incorporated at the C1 site, indicating the presence of short-range spatial interactions (Fig. S20). The electron–hole distributions of FOQ, FSQ, and FSeQ in the excited state revealed that the emission core remains localized within the N/C=O fused ring plane, which is essential for retaining the MR emission properties. Analysis based on transition density matrix heat maps indicates a high degree of similarity in the excited states of the three molecules, suggesting that the spatial noncovalent interactions represent a weak orbital interaction that has minimal influence on the electronic structure of the emission core (Fig. S25). The small values obtained from RMSD and *λ* calculations suggest that these three molecules are likely to exhibit narrow emission spectra (Table S7). In Fig. 5a, taking FSeQ as an example, mode-resolved decomposition of *λ* indicates that the modification effectively enhances molecular rigidity, with only minor contributions from low-frequency vibrations. Contributions from high-frequency vibrations account for 5–10 meV of *λ*, suggesting the presence of shoulder peaks in the emission. Fig. 5b further shows that the main transition dominates over other transitions in simulated spectra, pointing to a narrowing of the emission profile and improved color purity. The heat map of the Duschinsky matrix indicates that the vibration modes between the ground state and the excited state are less mixed in the low-frequency region (Fig. 5c). When plotted on the CIE chromaticity diagram, OQ, SQ, and SeQ exhibit CIE*y* coordinates around 0.6, corresponding to green emission. In contrast, SSeQ and FSeQ achieve CIE*y* coordinates of 0.047, meeting the BT.2020 standard, thus identifying them as promising candidates for high-purity display applications (Fig. S29).

**Fig. 6:**
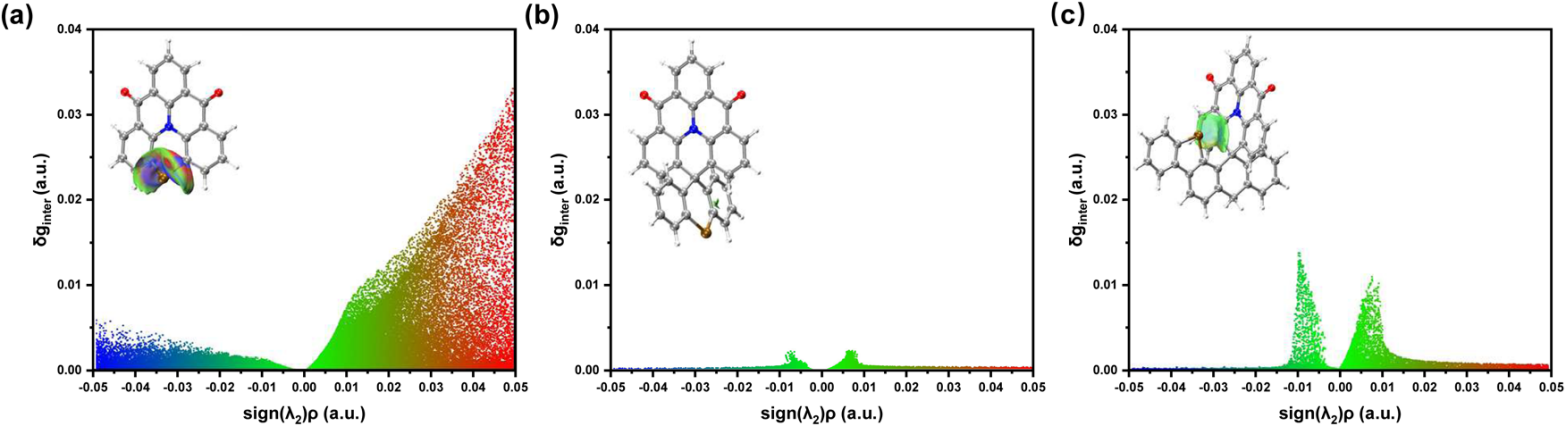
Independent gradient model based on Hirshfeld partition analyzes the interaction between the Se atom and the luminescent core in (a) SeQ, (b) SSeQ, and (c) FSeQ.

However, unlike the SSeQ molecule, the close-range heavy-atom effect in FSeQ significantly enhances the orbit SOC between singlet and triplet states. Specifically, 〈S|*Ĥ*_SO_|T〉|^2^ is only 0.13 cm^−1^ for SSeQ, whereas it increases to 3.24 cm^−1^ in FSeQ. IGMH analyzes shows three types of molecule have different degree of interaction between the Se atom and the luminescent core (Fig. 6). SeQ exhibits strong bonding interactions, while in SSeQ, the Se atom shows almost no interaction with the luminescent core. In contrast, FSeQ displays prominent short-range interactions characteristic of van der Waals forces. These interactions originate from the dynamic polarization and coupling between the p orbital electron cloud of the heavy atoms and the π orbital of the luminescent core , which can be fundamentally interpreted as electronic interactions between different orbitals. To further quantify the interaction between the heavy atoms and the luminescent core, we performed an energy decomposition analysis on the orbital interaction energies (Fig. S30). The results reveal distinct strengths across the three molecules: In SeQ, the Se atom engages in a typical bonding interaction with the core, yielding an orbital interaction energy more than −600 kcal mol^−1^. In contrast, SSeQ exhibits a negligible orbital interaction of only −1.5 kcal mol^−1^. Notably, FSeQ shows an orbital interaction energy of −23.4 kcal mol^−1^, which can be attributed to a through-space interaction between the heavy atoms and the luminescent core. This spatial interaction of heavy-atom effectively promotes singlet–triplet exciton coupling. Additionally, a comparative analysis of orbital interaction energies for the oxygen- and sulfur-containing series is provided in Fig. S30.

To further elaborate, the increase in SOC by heavy atoms is due to their higher nuclear charge, which results in a stronger Coulomb potential field. Electrons moving rapidly in this field experience pronounced relativistic effects, leading to a substantial enhancement of the SOC potential *ζ*(*r*). In particular, when the electron density distribution strongly overlaps with the spatial location of the heavy atom, the interaction between orbital and spin angular momenta becomes more pronounced, thereby improving the efficiency of singlet–triplet coupling. Such localized orbital–spin interactions effectively accelerate the upconversion of singlet to triplet excitons, ultimately enhancing the RISC process.

Notably, the SOC values of FSQ and FOQ are larger than those of OQ and SQ, as shown in Table 1. After careful analysis, we found that the OQ and SQ molecules possess high point group symmetry: OQ belongs to the *C*_2v_ point group, while SQ has Cs point group symmetry. This symmetry reduces the coupling strength between the S_1_ and T_1_ states, thereby leading to negligible SOC values.^63,64^ In contrast, FOQ and FSQ have larger molecular sizes, making it more difficult to maintain symmetry, thereby resulting in larger SOC values. This finding suggests that enhancing singlet–triplet SOC can be achieved not only through the heavy-atom effect but also by lowering molecular symmetry.^65,66^

## 4 Conclusions

This study systematically investigates the SOC and optical physics of three classes of MR-TADF molecules featuring QAO as the luminescent core with embedded heavy atoms, based on high-level quantum chemical calculations. Our computational results show good agreement with existing experimental data, thereby validating the reliability of the employed methodologies. Through an analysis of the excited-state properties, we revealed that the direct embedding of heavy atoms into the luminescent core introduces LRCT character into the excited state. This leads to enhanced relaxation effects and stronger electron-vibration coupling, resulting in a noticeable spectral red-shift and broadening. Comparative analyses among the three molecular classes demonstrate that the SOC enhancement induced by heavy-atom incorporation is highly localized, and its magnitude strongly depends on the specific position of the heavy atom within the molecular framework. In particular, when heavy atoms are positioned near the luminescent core or participate in frontier orbitals of the excited states, their contribution to SOC is markedly pronounced. Conversely, when they are distant from the excited-state distribution, the SOC enhancement is limited. Based on our findings, we summarize two key design guidelines for high-performance MR-TADF emitters:

(i) Homogenize SRCT for high color purity: a homogenized SRCT character in the S_1_ state leads to a more uniform signal in the transition density matrix heatmap. This homogeneity is crucial as it minimizes the potential energy surface displacement between the excited and ground states, thereby suppressing electron-vibration coupling during emission and ultimately enhancing color purity.

(ii) Strategic heavy-atom placement to balance SOC and color purity: the influence of heavy atoms on the excited state is highly dependent on their spatial position. Introducing heavy atoms at non-covalent sites adjacent to the key frontier molecular orbitals represents an effective strategy. This approach significantly enhances SOC while preserving the inherent SRCT characteristics of the luminescent core, thereby striking a balance between an accelerated RISC process and high color purity.

This study deepens our understanding of the mechanistic role of heavy-atom modification in MR-TADF systems, providing valuable theoretical insights and design guidelines for the development of narrowband MR-TADF emitters that combine both high *k*_RISC_ and excellent color purity.

## Author contributions

Shi-Jie Ge conceptualized the research, curated the data, and wrote the manuscript under the guidance of Zuo-Quan Jiang. Jian-Rong Wu contributed to the experimental discussion. Funding acquisition was done by Zuo-Quan Jiang.

## Conflicts of interest

The authors declare no conflict of interest.

## Supplementary Material

SC-OLF-D6SC00582A-s001

## Data Availability

The data supporting this article have been included as part of the supplementary information (SI). Supplementary information is available. See DOI: https://doi.org/10.1039/d6sc00582a.
