# Supplementary material for: Theoretical insights into site-specific heavy-atom effects on MR-TADF emitters: modulation of spin–orbit coupling and color purity
Source: Chem Sci. 2026 Mar 20;17(17):8493–503. doi: 10.1039/d6sc00582a (PMC13003568; doi:10.1039/d6sc00582a)
Supplement: SC-017-D6SC00582A-s001 [file SC-017-D6SC00582A-s001.pdf]

Supplementary Information (SI) for Chemical Science.
This journal is © The Royal Society of Chemistry 2026

Supporting Information

Theoretical Insights into Site-Specific Heavy-Atom Effects on MR-TADF Emitters: Modulation of Spin-Orbit Coupling and Excited-State Properties

*Shi-jie Ge, Jian-Rong Wu, Zuo-quan Jiang**

Table of Contents

1. **Computational results for OQ, SQ, and SeQ 2**
2. **Computational results for SOQ, SSQ, and SSeQ 7**
3. **Computational results for FOQ, FSQ, and FSeQ 11**
4. **The ω value obtained through LC-BLYP regulation 16**

1. Computational results for OQ, SQ, and SeQ

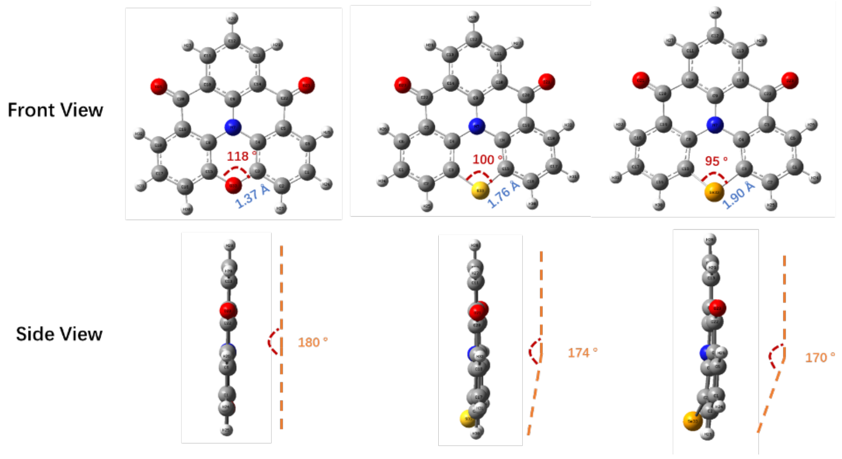

Fig. S1. The optimized ground-state structures of OQ, SQ, and SeQ, (top) front view, (bottom) side view. The front view includes the measurement of the C-X bond length and the C-X-C bond angle, while the side view includes the C-N-X bond angle measured along the central line to reflect the degree of out-of-plane bending of the molecule.

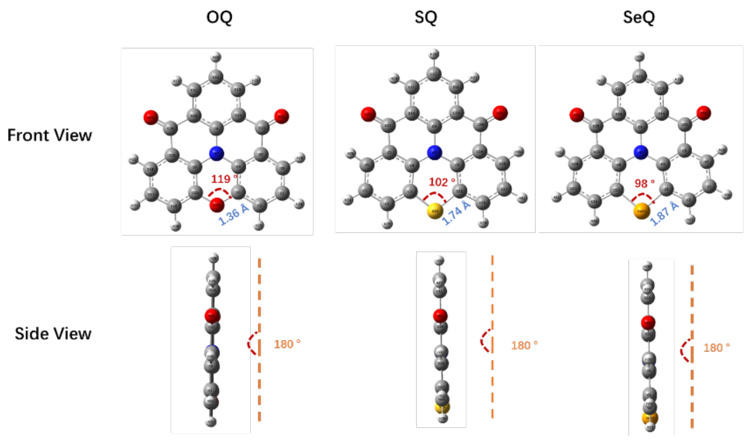

Fig. S2. The optimized excited-state (S_1) structures of OQ, SQ, and SeQ, (top) front view, (bottom) side view. The front view includes the measurement of the C-X bond length and the C-X-C bond angle, while the side view includes the C-N-X bond angle measured along the central line to reflect the degree of out-of-plane bending of the molecule.

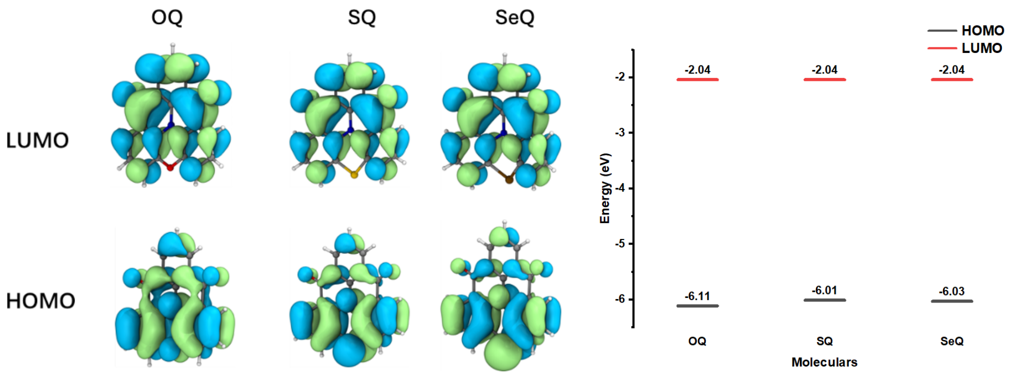

Fig. S3. Frontline molecular orbitals and energy leves of OQ, SQ, and SeQ.

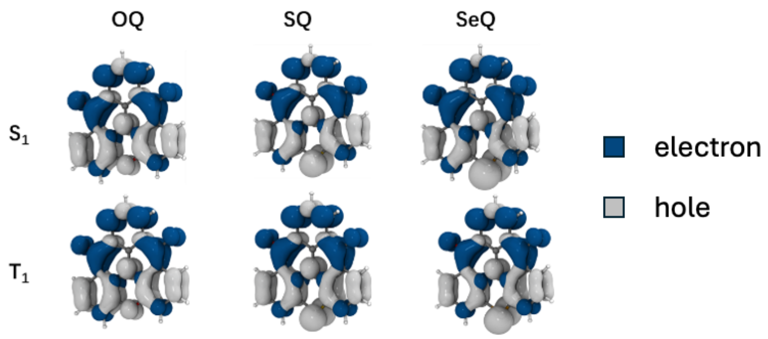

Fig. S4. Electron-hole distribution of the excited states of OQ, SQ, and SeQ.

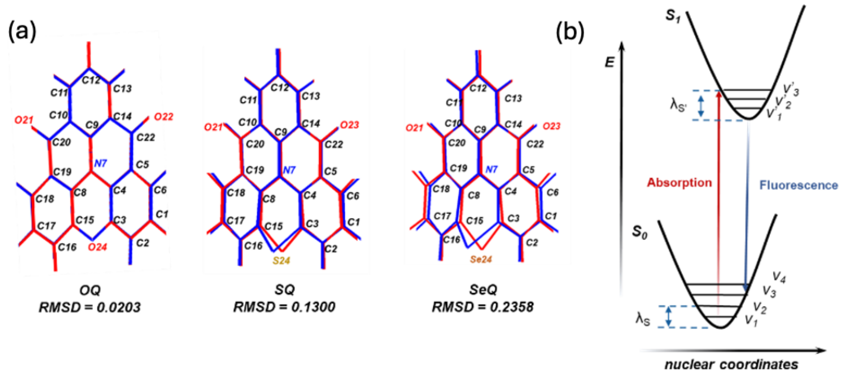

Fig. S5. (a) The geometric difference between the S_0 (blue) and S_1 (red) configurations for OQ, SQ, and SeQ; (b) Potential energy surfaces in the ground and the excited states.

Table S1. The root means square deviation (RMSD) values, reorganization energy and simulation of Stokes shifts for OQ, SQ,and SeO.

	**OQ**	**SQ**	**SeQ**
**RMSD**	0.02	0.13	0.24
$\lambda_{S'}$ **(eV)**	0.10	0.15	0.18
λ_{S} **(eV)**	0.11	0.12	0.14
$\lambda_{S'}+\lambda_{S}$ **(eV)**	0.21	0.27	0.32

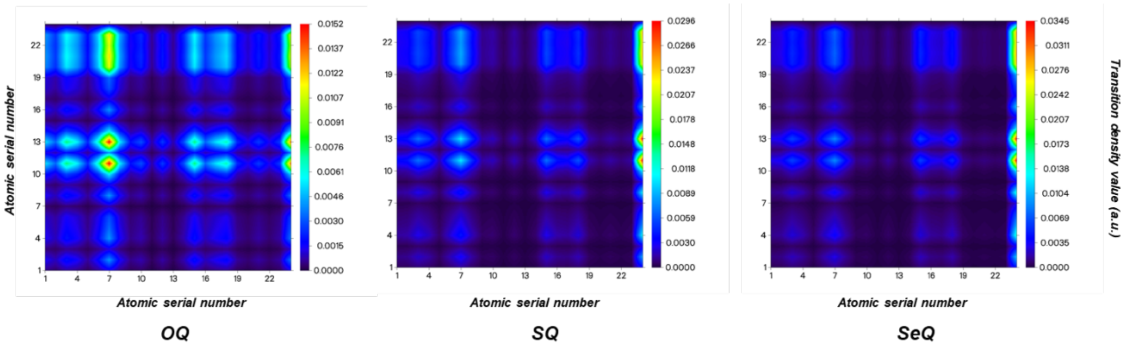

Fig. S6. Transition density matrix heat maps of OQ, SQ, and SeQ.

Table S2. Calculated **vertical excitation states** for the first three **singlet states** of molecules, with oscillator strengths (f), electron-hole center distances (D_idx), electron-hole overlap integrals (Sr), and orbital composition (orb) for each state.

	sn	*E* (eV)	*E* (nm)	f	D_idx (Å)	Sr (a.u.)	orb
**OQ**	1	2.92	425.2	0.166	1.454	0.594	H-L:0.972
	2	3.03	408.6	0.000	0.273	0.448	H3-L:0.718;H2-L:0.189
	3	3.20	387.0	0.000	0.532	0.465	H4-L:0.872;H3-L1:0.093
**SQ**	1	2.55	487.0	0.140	2.721	0.522	H-L:0.969
	2	3.14	394.4	0.000	0.555	0.456	H1-L:0.913;H3-L1:0.075
	3	3.30	375.6	0.000	0.610	0.464	H3-L:0.848;H1-L1:0.132
**SeQ**	1	2.579	480.8	0.122	3.01	0.489	H-L:0.963
	2	3.142	394.6	0.000	0.571	0.459	H2-L:0.908;H3-L1:0.077
	3	3.297	376.0	0.000	0.541	0.542	H3-L:0.755;H2-L1:0.125

Table S3. Calculated **adiabatic excitation states** for the first three **singlet states** of molecules, with oscillator strengths (f), electron-hole center distances (D_idx), electron-hole overlap integrals (Sr), and orbital composition (orb) for each state.

	sn	*E* (eV)	*E* (nm)	f	D_idx (Å)	Sr (a.u.)	orb
**OQ**	1	2.44	508.37	0.17	2.27	0.61	H-L:0.983;H3-L2:0.008
	2	3.07	404.02	0.00	0.17	0.45	H1-L:0.924;H2-L1:0.067
	3	3.20	386.89	0.00	0.21	0.47	H2-L:0.887;H1-L1:0.103
**SQ**	1	2.28	544.55	0.12	2.86	0.55	H-L:0.975;H-L2:0.012
	2	3.05	406.70	0.00	0.23	0.46	H1-L:0.917;H3-L1:0.073
	3	3.17	391.14	0.01	0.36	0.66	H-L2:0.977;H-L:0.012
**SeQ**	1	2.26	548.47	0.10	3.01	0.53	H-L:0.975;H-L2:0.013
	2	3.04	407.97	0.00	0.25	0.46	H2-L:0.914;H3-L1:0.075
	3	3.12	397.60	0.04	1.57	0.74	H-L1:0.965;H5-L2:0.01

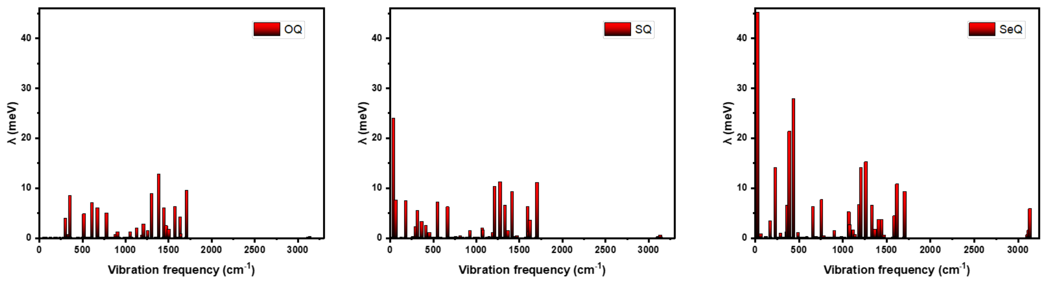

Fig. S7. Vibrationally resolved decomposition of reorganization energy in OQ, SQ, and SeQ.

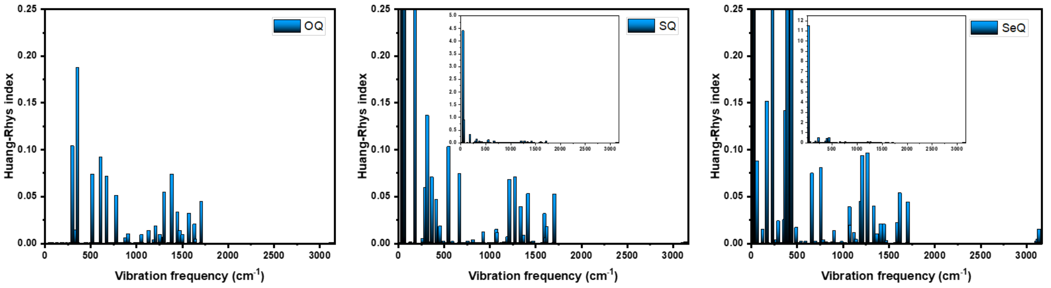

Fig. S8. Vibrationally resolved decomposition of Huang-Rhys factor in OQ, SQ, and SeQ.

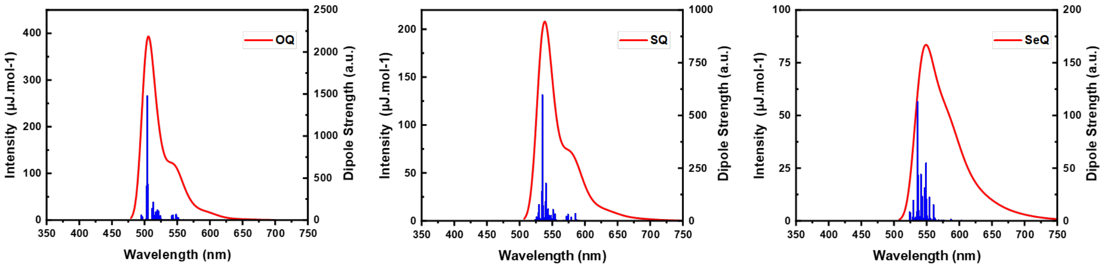

Fig. S9. Vibrationally resolved electronic spectra and different modes of vibronic coupling transitions.

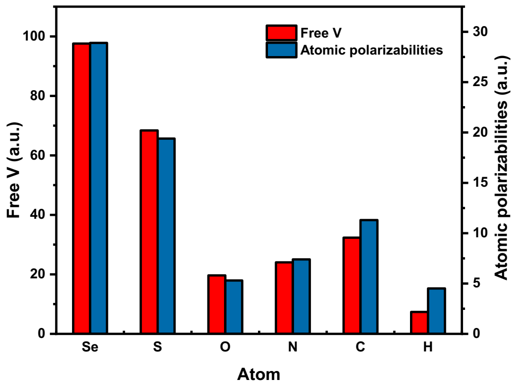

Fig. S10. Calculation of the free volume and polarizability of different atoms.

2. Computational results for SOQ, SSQ, and SSeQ

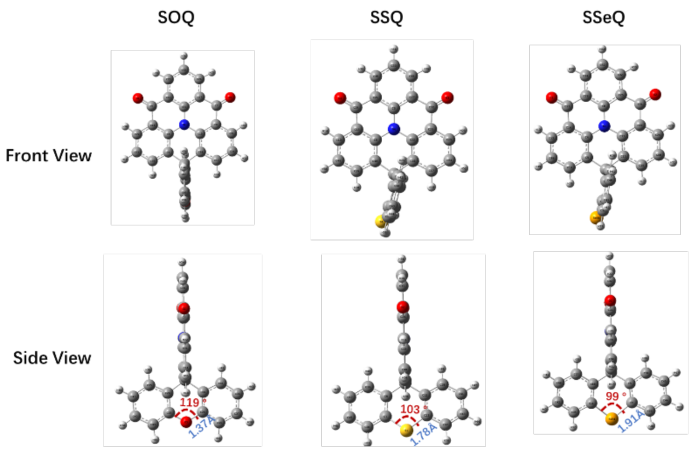

Fig. S11. The optimized ground-state structures of SOQ, SSQ, and SSeQ, (top) front view, (bottom) side view. The side view includes the measurement of the C-X bond length and the C-X-C bond angle.

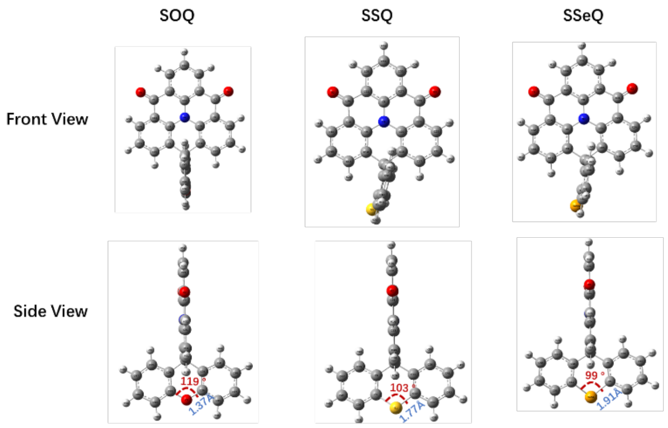

Fig. S12. The optimized excited-state (S_1) structures of SOQ, SSQ, and SSeQ, (top) front view, (bottom) side view. The side view includes the measurement of the C-X bond length and the C-X-C bond angle.

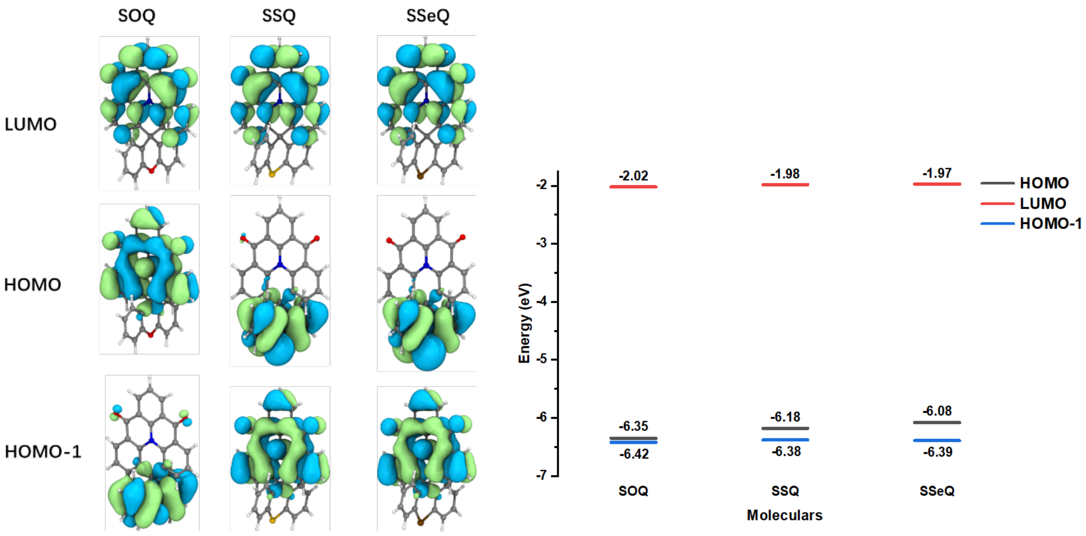

Fig. S13. Molecular orbitals and energy leves of SOQ, SSQ, and SSeQ.

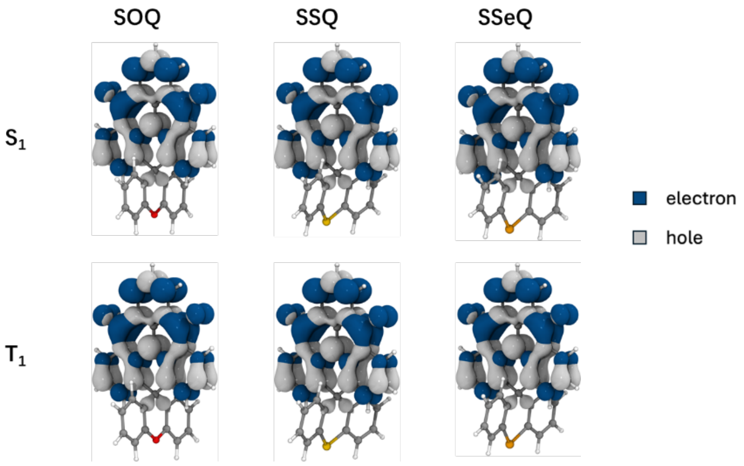

Fig. S14. Electron-hole distribution of the excited states of SOQ, SSQ, and SSeQ.

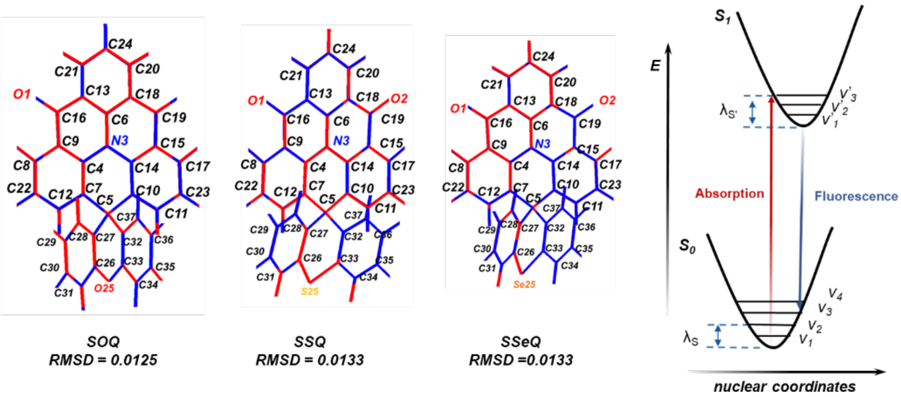

Fig. S15. (a) The geometric difference between the S_0 (blue) and S_1 (red) configurations for SOQ, SSQ, and SSeQ; (b) Potential energy surfaces in the ground and the excited states.

Table S4. The root means square deviation (RMSD) values, reorganization energy and simulation of Stokes shifts for SOQ, SSQ,and SSeO.

	**SOQ**	**SSQ**	**SSeQ**
**RMSD**	0.01	0.01	0.01
$\lambda_{S'}$ **(eV)**	0.08	0.08	0.08
λ_{S} **(eV)**	0.07	0.07	0.07
$\lambda_{S'}+\lambda_{S}$ **(eV)**	0.15	0.15	0.15

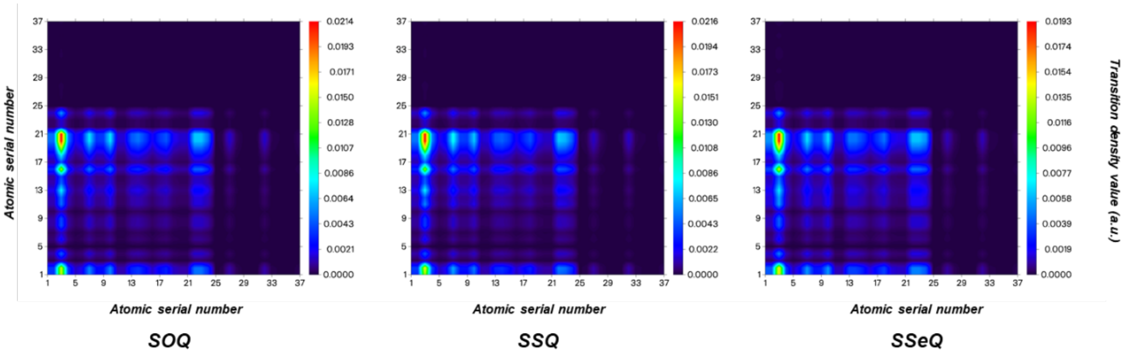

Fig. S16. Transition density matrix heat maps of SOQ, SSQ, and SSeQ.

Table S5. Calculated **vertical excitation states** for the first three **singlet states** of SOQ, SSQ, and SSeQ, with oscillator strengths (f), electron-hole center distances (D_idx), electron-hole overlap integrals (Sr), and orbital composition (orb) for each state.

	**sn**	***E*** **(eV)**	***E*** **(nm)**	**f**	**D_idx (Å)**	**Sr (a.u.)**	**orb**
**SOQ**	1	2.96	418.7	0.177	1.375	0.596	H-L:0.971
	2	3.05	406.3	0.000	0.205	0.460	H2-L:0.831;H1-L:0.095
	3	3.22	384.8	0.000	0.586	0.464	H3-L:0.85;H2-L1:0.131
**SSQ**	1	2.99	414.3	0.180	1.391	0.595	H1-L:0.97
	2	3.08	402.7	0.000	1.232	0.414	H2-L:0.655;H-L:0.275
	3	3.18	390.2	0.000	5.196	0.236	H-L:0.695;H2-L:0.25
**SSeQ**	1	3.00	413.4	0.1813	1.388	0.596	H1-L:0.97
	2	3.05	406.3	0.000	4.684	0.292	H-L:0.728;H2-L:0.233
	3	3.14	395.3	0.000	1.729	0.407	H2-L:0.672;H-L:0.248

Table S6. Calculated **adiabatic excitation states** for the first three **singlet states** of SOQ, SSQ, and SSeQ, with oscillator strengths (f), electron-hole center distances (D_idx), electron-hole overlap integrals (Sr), and orbital composition (orb) for each state.

	**sn**	***E*** **(eV)**	***E*** **(nm)**	**f**	**D_idx (Å)**	**Sr (a.u.)**	**orb**

**SOQ**	1	2.81	440.50	0.15	1.87	0.62	H-L:0.975;H7-L3:0.008
	2	2.95	419.60	0.00	0.07	0.46	H2-L:0.843;H1-L:0.087
	3	3.11	399.14	0.00	0.25	0.47	H3-L:0.875;H2-L1:0.11
**SSQ**	1	2.85	435.33	0.15	1.8	0.62	H1-L:0.974;H7-L3:0.008
	2	2.98	415.44	0.00	1.31	0.42	H2-L:0.67;H-L:0.261
	3	3.08	402.24	0.00	4.79	0.28	H-L:0.706;H2-L:0.23
**SSeQ**	1	2.86	434.21	0.15	1.84	0.62	H1-L:0.974;H7-L3:0.008
	2	2.96	419.15	0.00	4.56	0.31	H-L:0.724;H2-L:0.24
	3	3.04	407.75	0.00	1.83	0.42	H2-L:0.665;H-L:0.255

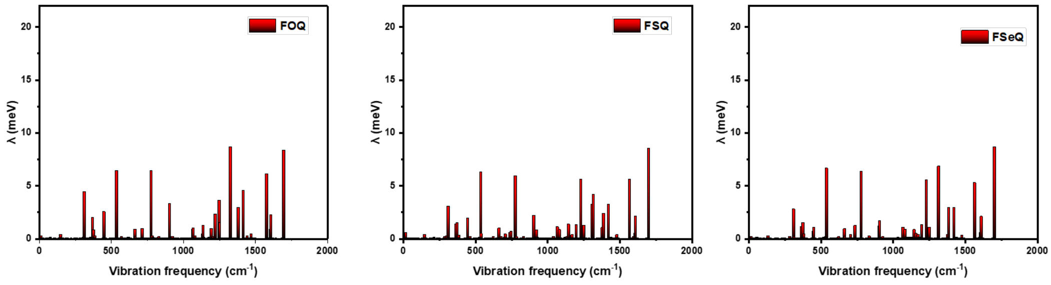

Fig. S17. Vibrationally resolved decomposition of reorganization energy in SOQ, SSQ, and SSeQ.

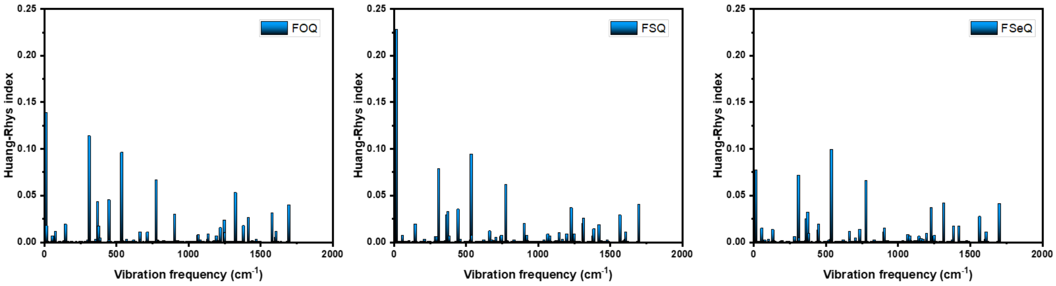

Fig. S18. Vibrationally resolved decomposition of Huang-Rhys factor in SOQ, SSQ, and SSeQ.

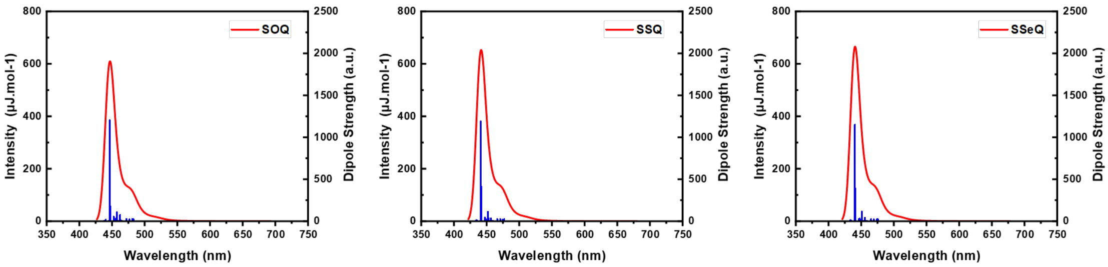

Fig. S19. Vibrationally resolved electronic spectra and different modes of vibronic coupling transitions.

3. Computational results for FOQ, FSQ, and FSeQ

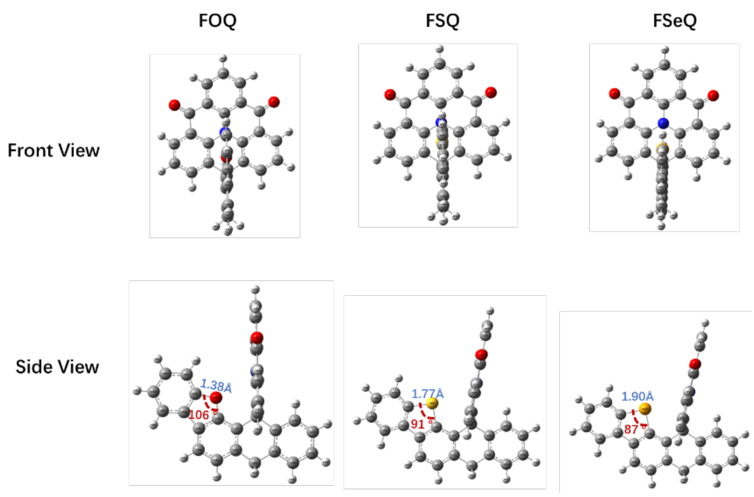

Fig. S20. The optimized ground-state structures of FOQ, FSQ, and FSeQ, (top) front view, (bottom) side view. The side view includes the measurement of the C-X bond length and the C-X-C bond angle.

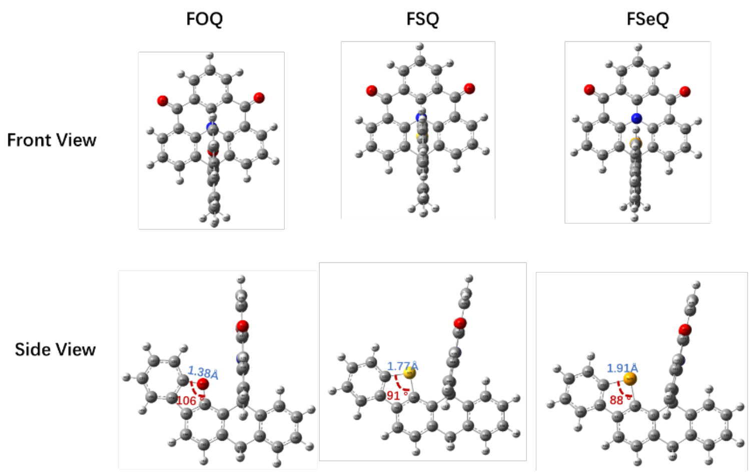

Fig. S21. The optimized excited-state (S_1) structures of FOQ, FSQ, and FSeQ, (top) front view, (bottom) side view. The side view includes the measurement of the C-X bond length and the C-X-C bond angle.

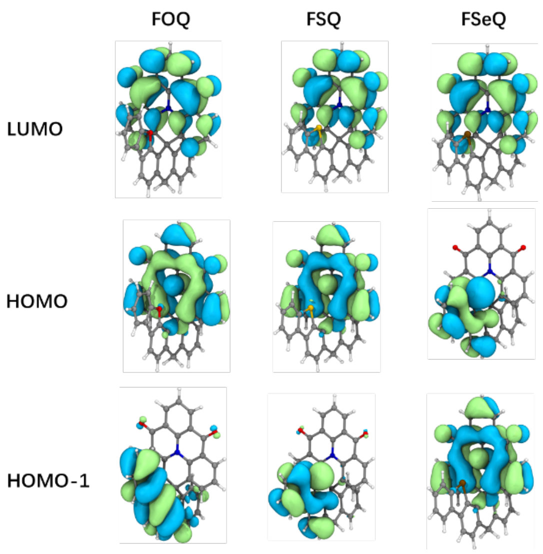

Fig. S22. Molecular orbitals and energy leves of FOQ, FSQ, and FSeQ.

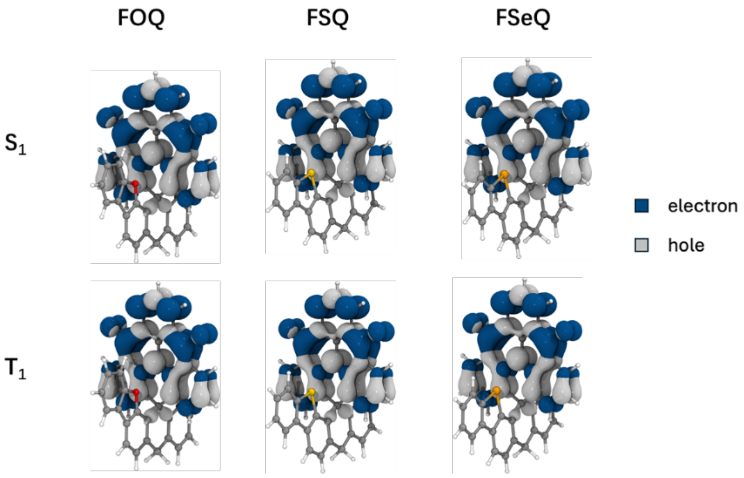

Fig. S23. Electron-hole distribution of the excited states of FOQ, FSQ, and FSeQ.

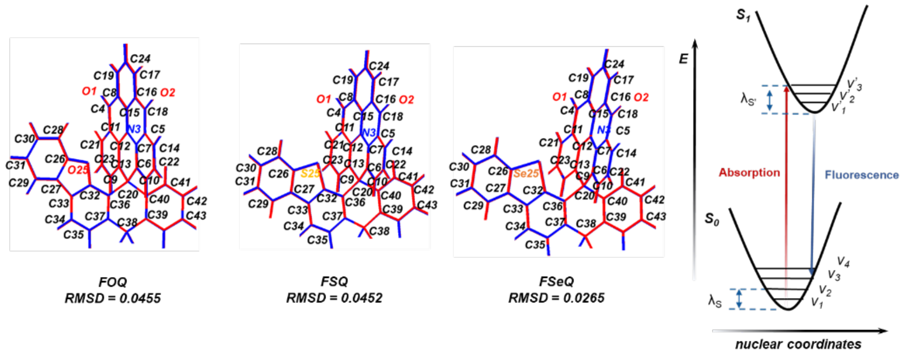

Fig. S24. (a) The geometric difference between the S_0 (blue) and S_1 (red) configurations for FOQ, FSQ, and FSeQ; (b) Potential energy surfaces in the ground and the excited states.

Table S7. The root means square deviation (RMSD) values, reorganization energy and simulation of Stokes shifts for FOQ, FSQ,and FSeO.

	FOQ	FSQ	FSeQ
**RMSD**	0.045	0.045	0.027
**$\lambda_{S'}$ (eV)**	0.093	0.087	0.083
**λ_S (eV)**	0.076	0.072	0.070
**$\lambda_{S'}$+λ_S (eV)**	0.169	0.159	0.153

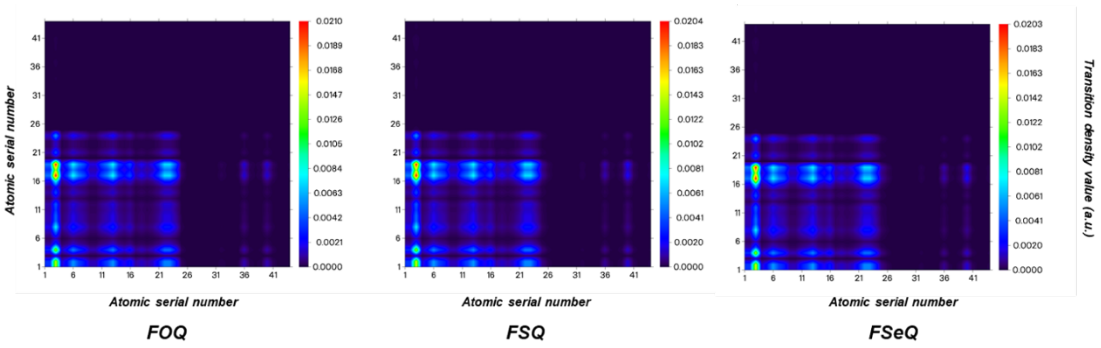

Fig. S25. Transition density matrix heat maps of FOQ, FSQ, and FSeQ.

Table S8. Calculated **vertical excitation states** for the first three **singlet states** of FOQ, FSQ, and FSeQ, with oscillator strengths (f), electron-hole center distances (D_idx), electron-hole overlap integrals (Sr), and orbital composition (orb) for each state.

	sn	*E* (eV)	*E* (nm)	f	D_idx (Å)	Sr (a.u.)	orb
**FOQ**	1	2.92	425.2	0.166	1.454	0.594	H-L:0.972
	2	3.03	408.6	0.000	0.273	0.448	H3-L:0.718;H2-L:0.189
	3	3.20	387.0	0.000	0.532	0.465	H4-L:0.872;H3-L1:0.093
**FSQ**	1	2.95	420.7	0.171	1.409	0.596	H-L:0.971
	2	3.06	405.8	0.000	0.245	0.456	H3-L:0.827;H5-L1:0.062
	3	3.22	384.5	0.000	0.592	0.459	H5-L:0.854;H3-L1:0.126
**FSeQ**	1	2.98	416.7	0.174	1.4	0.596	H1-L:0.971
	2	3.05	406.3	0.005	3.299	0.339	H-L:0.617;H3-L:0.319
	3	3.11	398.2	0.004	2.225	0.383	H3-L:0.566;H-L:0.359

Table S9. Calculated **adiabatic excitation states** for the first three **singlet states** of FOQ, FSQ, and FSeQ, with oscillator strengths (f), electron-hole center distances (D_idx), electron-hole overlap integrals (Sr), and orbital composition (orb) for each state.

	**sn**	***E* (eV)**	***E* (nm)**	**f**	**D_idx (Å)**	**Sr (a.u.)**	**orb**
**FOQ**	1	2.75	451.29	0.132	2.009	0.613	H-L:0.977
	2	2.93	422.57	0.000	0.096	0.459	H3-L:0.785
	3	3.08	402.33	0.000	0.196	0.469	H4-L:0.896
**FSQ**	1	2.79	444.42	0.139	1.924	0.621	H-L:0.976
	2	2.96	419.19	0.000	0.126	0.455	H3-L:0.837
	3	3.11	399.01	0.000	0.228	0.467	H5-L:0.879
**FSeQ**	1	2.81	439.11	0.141	1.878	0.625	H1-L:0.975
	2	2.95	419.68	0.005	3.269	0.352	H-L:0.635
	3	3.02	411.08	0.003	2.051	0.406	H3-L:0.582

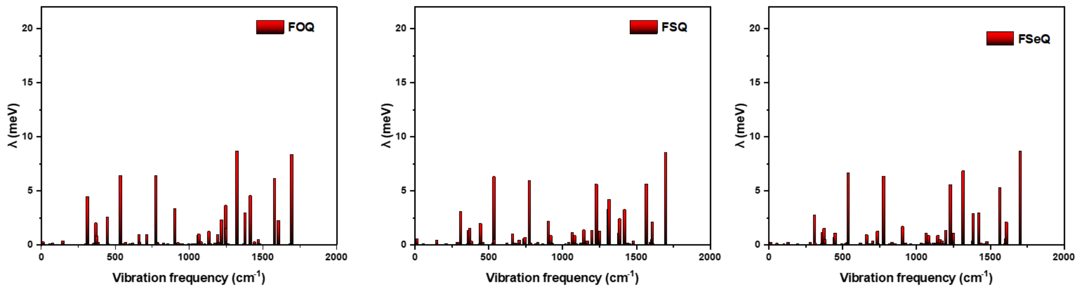

Fig. S26. Vibrationally resolved decomposition of reorganization energy in FOQ, FSQ, and FSeQ.

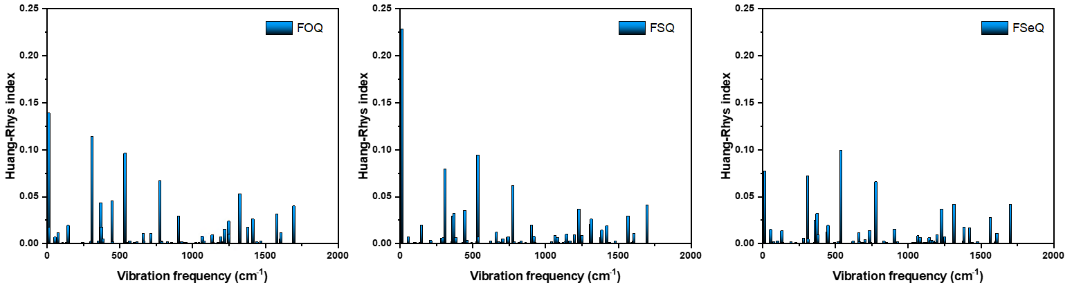

Fig. S27. Vibrationally resolved decomposition of Huang-Rhys factor in FOQ, FSQ, and FSeQ.

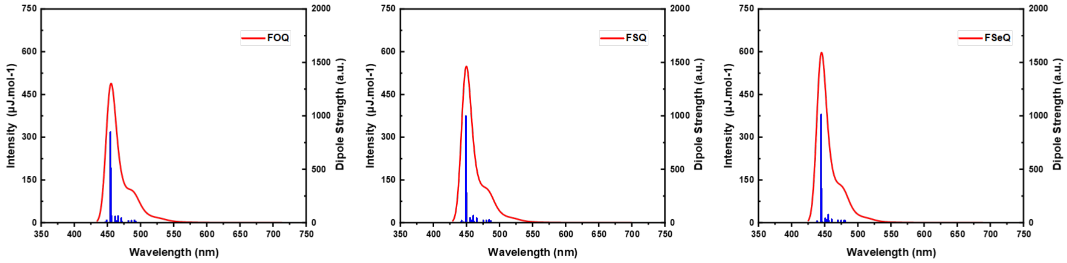

Fig. S28. Vibrationally resolved electronic spectra and different modes of vibronic coupling transitions.

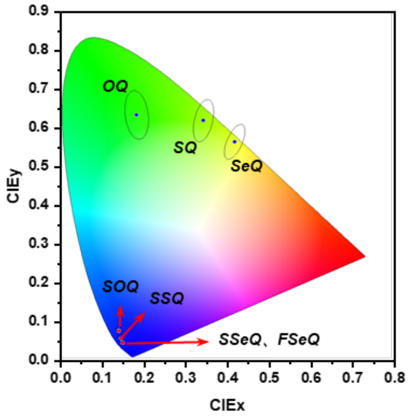

Fig. S29. The CIE coordinates based on the simulated spectrum

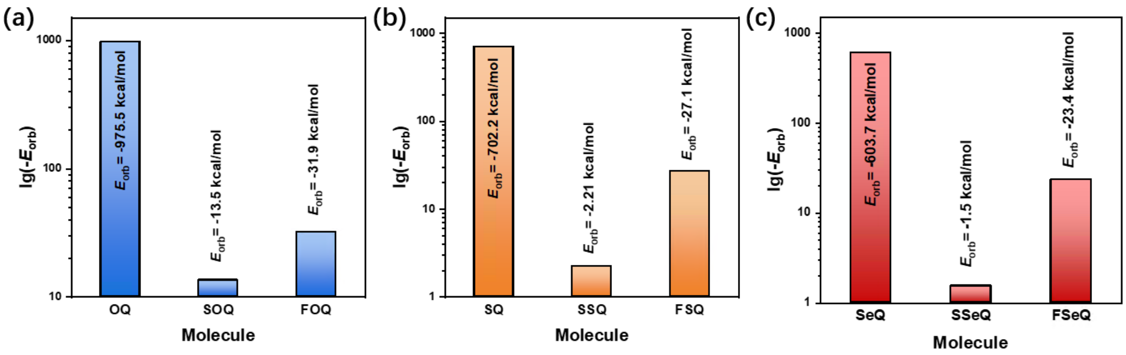

Fig. S30. The orbital interaction energies between heavy atoms and the luminescent core obtained through energy decomposition analysis of (a) oxygen series, (b) sulfur series and (c) selenium series

4. The ω value obtained through LC-BLYP regulation

Table S10. The ω value obtained through LC-BLYP regulation

	ω value
FOQ	0182600000
FSeQ	0200000000
FSQ	0191100000
OQ	0218800000
SeQ	0218300000
SOQ	0190000000
SQ	0218400000
SSeQ	0206200000
SSQ	0202900000
